# Genomic Analysis of Fluoroquinolone- and Tetracycline-Resistant *Campylobacter jejuni* Sequence Type 6964 in Humans and Poultry, New Zealand, 2014–2016

**DOI:** 10.3201/eid2512.190267

**Published:** 2019-12

**Authors:** Nigel P. French, Ji Zhang, Glen P. Carter, Anne C. Midwinter, Patrick J. Biggs, Kristin Dyet, Brent J. Gilpin, Danielle J. Ingle, Kerry Mulqueen, Lynn E. Rogers, David A. Wilkinson, Sabrina S. Greening, Petra Muellner, Ahmed Fayaz, Deborah A. Williamson

**Affiliations:** Massey University, Palmerston North, New Zealand (N.P. French, J. Zhang, A.C. Midwinter, P.J. Biggs, L.E. Rogers, D.A. Wilkinson, S.S. Greening, A. Fayaz);; New Zealand Food Safety Science and Research Centre, Palmerston North (N.P. French, D.A. Wilkinson);; The University of Melbourne, Melbourne, Victoria, Australia (G.P. Carter, D.J. Ingle, D.A. Williamson);; Institute of Environmental Science and Research Limited, Christchurch, New Zealand (K. Dyet, B.J. Gilpin);; Australian National University, Canberra, Australian Capital Territory, Australia (D.J. Ingle);; Poultry Industry Association of New Zealand, Auckland, New Zealand (K. Mulqueen);; EPI-interactive, Wellington, New Zealand (P. Muellner)

**Keywords:** *Campylobacter jejuni*, antimicrobial resistance, poultry, plasmid, tetracycline, fluoroquinolone, bacteria, New Zealand

## Abstract

In 2014, antimicrobial drug–resistant *Campylobacter jejuni* sequence type 6964 emerged contemporaneously in poultry from 3 supply companies in the North Island of New Zealand and as a major cause of campylobacteriosis in humans in New Zealand. This lineage, not previously identified in New Zealand, was resistant to tetracycline and fluoroquinolones. Genomic analysis revealed divergence into 2 major clades; both clades were associated with human infection, 1 with poultry companies A and B and the other with company C. Accessory genome evolution was associated with a plasmid, phage insertions, and natural transformation. We hypothesize that the *tetO* gene and a phage were inserted into the chromosome after conjugation, leaving a remnant plasmid that was lost from isolates from company C. The emergence and rapid spread of a resistant clone of *C. jejuni* in New Zealand, coupled with evolutionary change in the accessory genome, demonstrate the need for ongoing *Campylobacter* surveillance among poultry and humans.

Campylobacteriosis caused by *Campylobacter jejuni* is one of the most common zoonotic diseases; in many countries, incidence is increasing (*1*). Typically, human infection with *C. jejuni* results in an acute, self-limiting gastroenteritis, and treatment is largely supportive. However, antimicrobial drug treatment is indicated for patients who have invasive infection, have severe and persistent gastroenteritis, or are immunocompromised. The mainstays of therapy are macrolides and fluoroquinolones; however, resistance to these drugs, particularly fluoroquinolones, is common in many parts of the world and precludes their clinical usefulness (*2*).

Among industrialized countries, one of the highest rates of campylobacteriosis is found in New Zealand. In 2017, there were 6,482 notified cases in New Zealand, representing an incidence of ≈150 cases/100,000 population (*3*). The high proportion of cases in New Zealand is thought to result from ingestion of contaminated food, typically undercooked poultry, which has prompted regulatory and voluntary implementation of control measures along the poultry supply chain (*4*).

Poultry production in New Zealand is dominated by 3 major supply companies and several smaller companies. No fresh chicken meat is imported into New Zealand (*5*). Studies in New Zealand have identified dominant multilocus sequence types (STs) of *C. jejuni* associated with poultry from particular companies; the most prevalent ST associated with human cases during 2005–2008, ST474, was predominant in poultry from 1 company (*5**–**7*). The vertically contained nature of the New Zealand poultry supply, which involves minimal transfer of birds between poultry companies, is considered to be a major contributor to the dominance of particular strains at individual companies (*5*).

In May 2014, a previously unreported *C. jejuni* clone of ST6964, a member of a poultry-associated clonal complex (CC), CC354 (*8*), resistant to fluoroquinolones and tetracyclines, was isolated from poultry carcasses at a *Campylobacter* sentinel surveillance site (*9*) on the North Island of New Zealand. Concurrently, throughout 2014 and 2015, sporadic and outbreak-associated human cases of campylobacteriosis associated with resistant ST6964 were identified across New Zealand. Subsequent cross-sectional studies of poultry and humans suggested that fluoroquinolone resistance in *C. jejuni* had increased from <5% to 19% over 1 year (*10*)*.* The unprecedented rapid emergence and geographic spread of this resistant strain has widespread implications. A marked shift from low to relatively high levels of antimicrobial drug resistance in *Campylobacter* spp. in New Zealand is a concern for food safety and public health. Furthermore, evidence of very rapid spread across the vertically contained poultry companies requires reevaluation of biosecurity measures in the industry. To determine which factors may have contributed to the dissemination of this clone in New Zealand, we undertook a detailed genomic analysis of ST6964 isolates from humans and poultry collected during 2014–2016.

## Methods

### Ethics Statement

Approval from the Multi-Region Ethics Committee, New Zealand, is provided for work related to the *Campylobacter* sentinel surveillance site (application no. MEC/10/16/EXP). Isolates were collected and analyzed through routine public health investigation activities. The 2006 guidelines from the National Ethics Advisory Committee, Ministry of Health, Wellington, New Zealand, state that these activities do not require ethics committee review.

### Setting and Sampling Strategies

The 2014 isolation of antimicrobial drug–resistant *C. jejuni* ST6964 from humans and poultry led to systematic surveys of cases in humans and poultry aimed at determining the extent of spread of this lineage into the food supply and the origin of human cases. During 2014–2016, we collected antimicrobial drug–resistant *C. jejuni* ST6964 isolates from 4 sources. The first source was whole poultry carcasses and fecal samples from humans with campylobacteriosis at the sentinel site from May 1, 2014, through December 31, 2015 (*9*). The second source was a cross-sectional survey of human cases conducted from May 1 through October 31, 2015 (*10*). Five community diagnostic laboratories, covering all major population centers in the North and South Islands, were asked to refer *C. jejuni* isolates from humans to the Institute of Environmental Science and Research Ltd (ESR), Wellington, New Zealand, for the survey. The third source was samples submitted directly to ESR from humans with antimicrobial drug–resistant campylobacteriosis. The fourth source was 2 cross-sectional studies of pooled cecal samples from slaughtered poultry from the major companies (Appendix Figure 1). A total of 227 isolates were included in the analysis.

### Microbiological Testing

We isolated *C. jejuni* from whole poultry carcasses and fecal samples from humans at the sentinel site as described previously (*11*). Isolates from pooled cecal samples came from swab samples taken from the pooled ceca of 5 chickens that were from the same poultry shed and slaughtered in a commercial poultry factory. Swabs in Amies with charcoal transport media (Copan, https://www.copangroup.com) were transported chilled to *^m^*EpiLab (Massey University, Palmerston North, New Zealand) for microaerobic culture at 42°C in a microaerobic incubator (Don Whitley Scientific, https://www.dwscientific.com) on modified charcoal cefoperazone deoxycholate agar (mCCDA; Fort Richard, https://www.fortrichard.com) and mCCDA-cip/tet (LabM Ltd., http://www.labm.com) containing 1 μg/mL ciprofloxacin and 4 μg/mL tetracycline (Sigma-Aldrich, https://www.sigmaaldrich.com). We subcultured colonies that resembled *C. jejuni* on the mCCDA-cip/tet plates onto Columbia horse blood agar plates (Fort Richard) and incubated them microaerobically at 42°C. All isolates from poultry carcasses and human fecal samples and a subset of those isolated from mCCDA-cip/tet from pooled chicken ceca were speciated by PCR (*12*). We determined susceptibility to ciprofloxacin and tetracycline according to Clinical and Laboratory Standards Institute (CLSI) methods, by using disk diffusion (*13*).

For human clinical isolates from the cross-sectional survey and direct submissions to ESR, we determined susceptibility to ciprofloxacin, erythromycin, and tetracycline by using the methods described by the CLSI, with either Etests or disk diffusion (*13*). Tests were performed on Mueller-Hinton agar with 5% sheep blood and incubated microaerobically at 36°C–37°C for 48 h. We interpreted MICs according to CLSI breakpoints (*13*) and disk-diffusion results according to European Committee on Antimicrobial Susceptibility Testing clinical breakpoints (*14*). We subtyped *C. jejuni* isolates from humans and fresh chicken carcasses and a subset of those from pooled chicken ceca by multilocus sequence typing (MLST) (*15*) as described (*11*).

### Whole-Genome Sequencing

For Illumina sequencing, we extracted genomic DNA from bacterial isolates on a JANUS automated workstation (PerkinElmer, https://www.perkinelmer.com) by using Chemagic magnetic bead technology, according to the manufacturer’s instructions. We prepared DNA libraries by using a NexteraXT DNA preparation kit (Illumina, https://www.illumina.com) and performed 2 × 100 bp sequencing on the NextSeq 500 platform (Illumina), as previously described (*16*). Four representative *C. jejuni* isolates also underwent whole-genome sequencing on the Pacific Biosciences, Inc., RS II platform (https://www.pacb.com). For this, genomic DNA was extracted from overnight cultures by using the Genelute bacterial genomic DNA kit (Sigma Aldrich). DNA libraries were prepared according to the 20 kb Template Preparation using the BluePippin DNA Size Selection system protocol (Pacific Biosciences, Inc). Sequence data are available from GenBank BioProject ID PRJNA520992 and PubMLST (https://pubmlst.org/campylobacter) nos. 70207–12, 70229, 70230, 70232, 70233, 70252, 70253, and 78631–845.

### Genome Assembly

For processing and quality control of the Illumina reads, we used QCtool pipeline (https://github.com/mtruglio/QCtool). To assemble the processed reads, we used the SPAdes genome assembler version 3.12.0 (*17*).

### Whole-Genome MLST Phylogeny

To define whole-genome MLST allelic profiles, we used Fast Genome Profiler (Fast-GeP, https://github.com/jizhang-nz/fast-GeP) (*18*) and the complete chromosome sequence of isolate 15AR0984 (generated in this study) as a reference. Phylogenetic relationships were displayed as NeighborNets by using SplitsTree 4 (*19*). The whole-genome polymorphic sites–based phylogeny was inferred from the concatenated sequences of the coding sequences shared by all the whole-genome sequences. We predicted and eliminated all regions with elevated densities of base substitutions and reconstructed the phylogenetic relationship of the remaining recombination-free sequences by using Gubbins version 2.3.4 with the default settings (*20*). We further examined the relationship by using the 1,343 genes in the *C. jejuni* core-genome MLST scheme version 1.0 (*21*) on the *Campylobacter* PubMLST website (https://pubmlst.org/campylobacter).

### Single-Nucleotide Polymorphism Phylogeny

We mapped 227 genomes to complete chromosome reference 15AR0984 (completed with PacBio sequencing, https://www.pacb.com) by using Snippy version 4.3.5 with mincov (the minimum number of reads covering a site to be considered) of 10 and minfrac (the minimum proportion of those reads that must differ from the reference) of 0.9 (https://github.com/tseemann/snippy). We filtered the resulting single-nucleotide polymorphism (SNP) alignment for recombination by using Gubbins (*20*), allowing for 50 iterations and specifying the weighted Robinson-Foulds convergence method. We extracted core SNPs by using SNP sites (*22*), giving a final total of 70 SNPs in the core genome. We then used the filtered alignment as input for IQtree (*23**,**24*) along with a general time reversible plus gamma 4 model, constant sites (606841, 268757, 264881, 606654), ultrafast bootstrapping with 1,000 replicates, and the SH-aLRT parameter with 1,000 bootstrap replicates to infer phylogenetic structure. We visualized the phylogeny in R (https://www.r-project.org) by using the package *ggtree* (*25*)*.* We investigated pairwise SNP distances by using HarrietR (https://github.com/andersgs/harrietr). We visualized the recombination regions detected in the 227 genomes from Gubbins (*20*) by using Phandango (*26*) and annotated the reference chromosome 15AR0984 using Prokka version 1.13 (*27*).

### Comparative Genomics of Mobile Elements

The reference genome 15AR0984 contained a plasmid (15AR0984-m) that was 43,680-bp long. We calculated the likelihood of this plasmid and other chromosomally integrated mobile elements being in each of the 227 genomes by using a method described previously (*28*). We considered the mobile elements CJIE1, CJIE2, CJIE3, and CJIE4 from the reference genome RM1221 (*29*) and a variant of CJIE1, named CJIE1v, which was also present in the reference genome 15AR0984. We plotted these data against the inferred phylogenetic tree in R by using *ggtree* (*25*). We examined the locations of chromosomally integrated mobile elements in the 4 PacBio complete genomes (15AR0984, 15AR0917, 15AR0919, and 15AR1555) and the reference strain RM1221 by using Mauve (*30*) and BLAST Ring Image Generator (*31*).

To find closely related plasmids to 15AR0984-m, we used the complete sequence as a query to BLAST (https://blast.ncbi.nlm.nih.gov/Blast.cgi) against the GenBank Nucleotide collection (nr/nt) database. We performed phylogenetic analyses of the most similar plasmids (Appendix Table 1) by using the whole-genome MLST method described and using the 15AR0984-m plasmid as the reference and presented as a NeighborNet using SplitsTree (*19*).

## Results

### Rapid Emergence of *C. jejuni* ST-6964 in Humans and Poultry

*C. jejuni* ST6964 with dual resistance to ciprofloxacin and tetracycline was first identified through sentinel surveillance in May 2014 in 2 retail poultry carcasses sampled in Palmerston North, Manawatu, New Zealand. The only other members of CC354 identified in the country to date are at least 2 locus variants of ST6964, according to the 7-gene MLST scheme, and only 1 other ST-6964 isolate has been reported outside of New Zealand, originating from China (https://pubmlst.org/campylobacter).

By July 2014, *C. jejuni* ST6964 had been identified in 3 poultry companies, and by August 2014, the first human case was observed at the sentinel site. A total of 3 (1.8%) of 165 human cases at the sentinel site were identified as being caused by ST6964 in 2014 and 4 (3.3%) of 122 human cases in 2015. A total of 10 (13.9%) of 72 retail poultry carcasses at the sentinel site were positive for ST6964 in 2014 and 25 (34.7%) of 72 in 2015. A total of 41 isolates from unique samples (7 human and 34 poultry) from the sentinel site underwent whole-genome sequencing and were included in this study.

In light of findings from the sentinel site, ESR conducted a national survey of antimicrobial-resistant *C. jejuni* in human patients in New Zealand during May–October 2015 (*10*). A total of 297 isolates were referred from 5 clinical laboratories: 3 on the North Island and 2 on the South Island. This survey provided 22 of the *C. jejuni* ST6964 isolates included in this study; 21 were from patients in the North Island and 1 was from a patient in the South Island. In addition to the survey, another 28 isolates from human patients on the North Island were included in this study from samples submitted directly to ESR from diagnostic laboratories.

To assess the extent of spread of *C. jejuni* ST6964 in poultry, we undertook 2 systematic surveys of poultry carcasses from slaughter plants servicing poultry companies A–D. Only samples from companies A, B, and C, which are based in the North Island, were positive for this ST; these companies accounted for 136 of the isolates included in this study.

All sequenced *C. jejuni* ST6964 isolates were confirmed as phenotypically resistant to ciprofloxacin and tetracycline. All tetracycline-resistant ST6964 isolates harbored the *tetO* gene, which was located at a previously described insertion site, between the *kdsB* and CJE0905 genes (*32*). The C257T (Thr86Ile) mutation in *gyrA,* associated with fluoroquinolone resistance (*33*), was present in all ciprofloxacin-resistant isolates.

### Relationship between Core Genomes of *C. jejuni* ST6964 from Humans and Poultry

**Figure 1:**
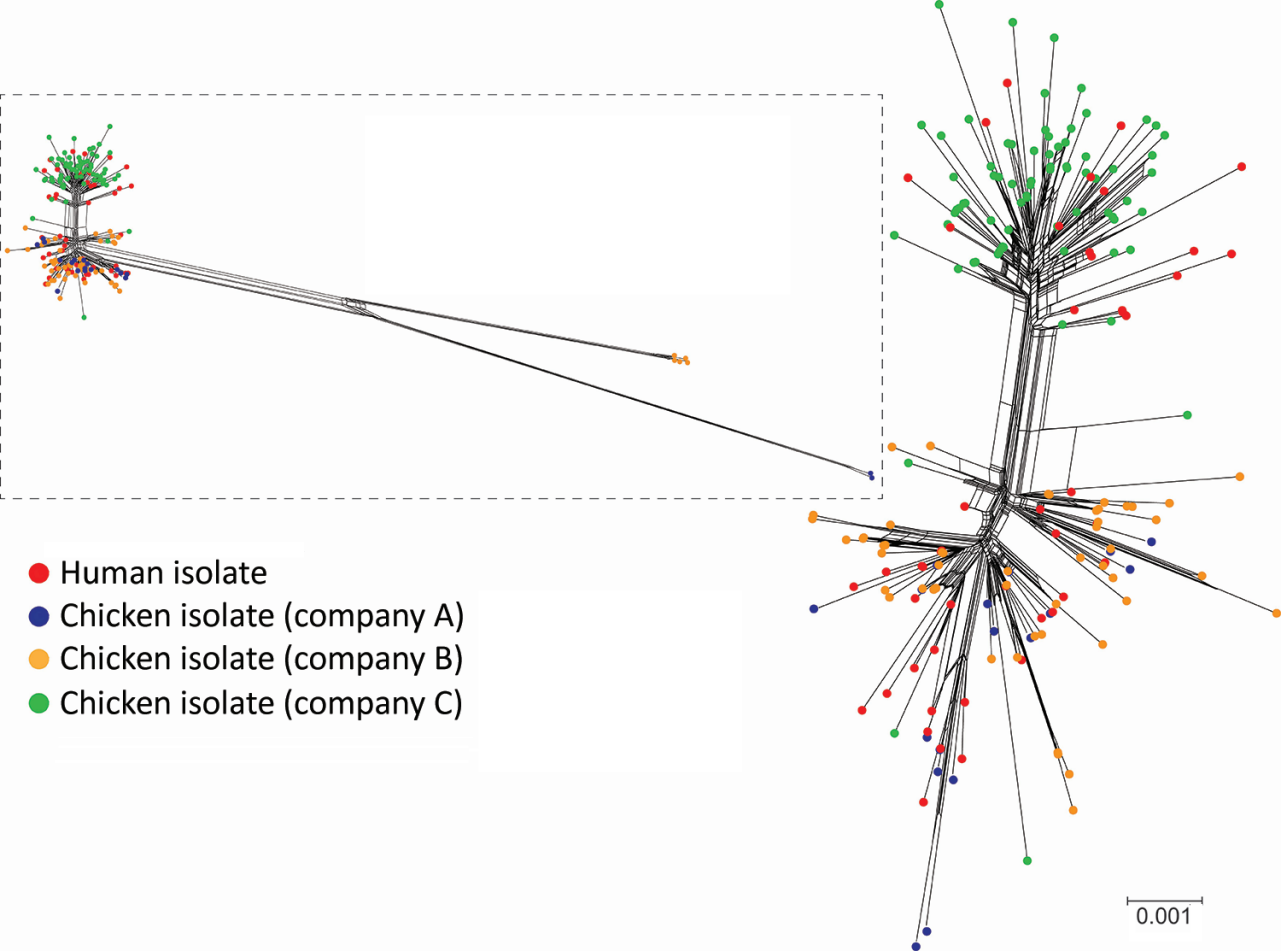
NeighborNet phylogenies generated from the allele profiles identified in the whole-genome multilocus sequence typing analysis of 227 sequence type 6964 *Campylobacter jejuni* isolates from humans and poultry, New Zealand, 2014–2016. The corrected NeighborNet network was generated after eliminating the 87 loci that were identified in predicted recombinant regions (Appendix Table 1). Inset shows the uncorrected NeighborNet network, generated with the original 1,363-loci allele profiles. Scale bar indicates the whole-genome multilocus sequence typing distance, which represents the number of allelic differences per shared locus.

We used 3 complementary approaches to assess relatedness of human and poultry isolates: whole-genome MLST using Fast-GeP (*18*), core-genome MLST using the *Campylobacter* PubMLST scheme (*21*), and SNP-based phylogeny. Fast-GeP analysis found 1,363 complete coding sequences that were single copy and shared by all 227 isolates. Most of the loci were identical across isolates (n = 1,163 loci), and NeighborNet distances and a NeighborNet network revealed 2 clades, 1 associated with poultry companies A and B and 1 with company C. A similar relationship was evident after removal of hypothetical recombination regions (Figure 1; Appendix Table 2). A similar NeighborNet profile and distribution among poultry companies resulted from the core-genome MLST results (Appendix Figure 2); 954 of the loci were identical and 389 were polymorphic.

**Figure 2:**
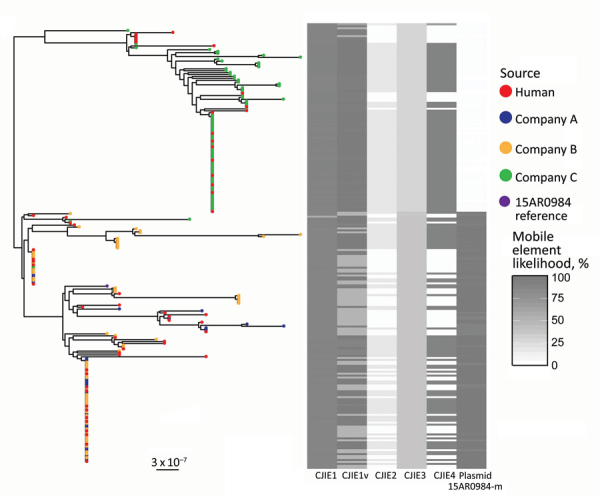
Population structure of 227 sequence type 6964 *Campylobacter jejuni* isolates from humans and poultry, New Zealand, 2014–2016. The tree is the inferred midpoint rooted phylogeny of the isolates, including the reference 15AR0984 genome. The tips are colored by source of the *C. jejuni* isolate. The heatmap indicates the likelihoods of the presence of mobile elements including CJIE1 variant (cjie1_15AR0984), CJIEs 1–4, and the plasmid 15AR0984-m. Dark shading on the heatmap indicates 100% likelihood; white indicates absence. Scale bar indicates nucleotide substitutions per site.

**Figure 3:**
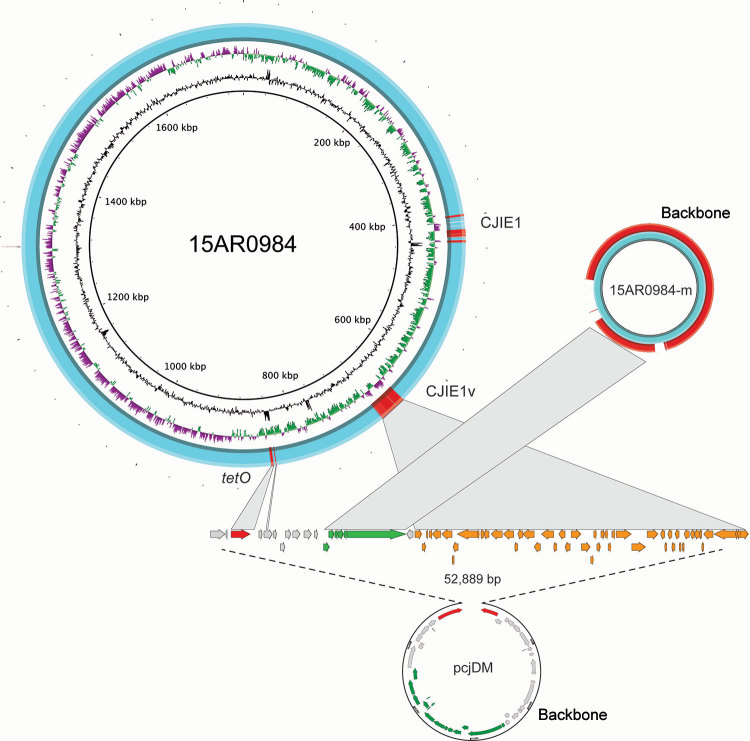
Genome structures of the complete *Campylobacter jejuni* strain 15AR0984 chromosome and plasmid (15AR0984-m) isolated from humans and poultry, New Zealand, 2014–2016, compared with the closest plasmid (pcjDM) sequence found in GenBank. High-scoring segment pairs between the 15AR0984 genome and the plasmid pcjDM ware connected with gray bars to illustrate the similar shared regions except for the backbone regions, which were highly conserved across the pTet-like plasmid genomes.

We constructed an SNP-based phylogeny after removal of recombinant regions (Figure 2) and identified recombinant block and associated genes (Appendix Table 1, Figure 3). We found a maximum of 13 SNPs between any single pair of isolates in the 70 shared-SNP loci present in nonrecombinant regions. The lower genetic diversity between isolates in the SNP analysis compared with the allele-based analysis was attributable to the removal of insertion and deletion mutations and loci subject to recombination. In the SNP analysis, isolates were again segregated into distinct clades strongly associated with poultry companies and carriage of mobile elements (Figure 2). Isolates from humans admixed with isolates from poultry in all clades, suggesting that the human infections were linked to poultry from all supply companies.

### Plasmid Sequences Associated with Distinct ST6964 Lineages

We identified high mobile element likelihood scores (>90) for plasmid 15AR0984-m in 131 (58%) of the isolates. Plasmid sequences were strongly associated with the core phylogeny and 2 of the 3 poultry companies, A and B (Figure 2). Plasmid 15AR0984-m showed high sequence and structural similarities with other previously described *tetO* carrying pTet plasmids and shares the same backbone as *tetO* plasmids pcjDM, S3, and pRM4661 (Figure 3; Appendix Table 1, Figure 4).

The plasmid 15AR0984-m was most closely related to the *tetO* megaplasmid pcjDM (Appendix Figure 4), which also contains a prophage (*34*) that shows sequence homology with integrated elements found in multiple ST6964 isolates. These elements are labeled CJIE1 and CJIE1v (a variant of CJIE1), and the latter was most similar to the prophage in plasmid pcjDM. Both prophage integrated elements bear similarities to integrated element CJIE1 identified in *C. jejuni* isolate RM1221 (*29*,*35*). All but 1 isolate (H2239a) contained CJIE1, and all were identified at the same location in the chromosome of the 4 isolates sequenced with PacBio. In contrast, 71% (162/227) of the isolates contained CJIE1v, as did 3 of the 4 complete genomes (15AR0984, 15AR0919, and 15AR1555). Although CJIE1 was located at the same chromosomal position in all 4 complete genomes, CJIE1v was located at a different position in the chromosome of 3 of the 4 that contained this mobile element (Appendix Figure 5). 

In addition to the 2 CJIE1-like elements, 65% (148/227) of genomes showed evidence of a previously described integrated element CJIE4 (*29*), located in the same chromosomal location in 2 of 4 complete genomes (15AR0984 and 15AR0919) and the reference genome *C. jejuni* RM1221 (*29*) (Appendix Figure 6). CJIE4 was identified in isolates from all poultry companies and in 26 of 57 isolates from humans. The *dns* gene (CJE0256), encoding for an extracellular desoxyribonuclease, was carried in CJIE1 in all but 1 of our isolates (H2239a). However, none of the CJIE1v elements contained the *dns* gene (CJE0256). Although 149 of 227 *C. jejuni* ST6964 isolates carried CJIE4, none of the CJIE4 elements had the DNA/RNA nonspecific endonuclease gene (CJE1441) present in the CJIE4 element of strain RM1221. 

## Discussion

Data from the 4 sources, (i.e., sentinel surveillance, human case survey, direct submission of samples from humans, and samples from poultry) demonstrated rapid emergence of a resistant lineage of *C. jejuni* among isolates from humans and poultry in New Zealand from 2014 on, indicating how rapidly national levels of resistance can change through the introduction of a successful bacterial clone. Despite high rates of campylobacteriosis in New Zealand, rates of *C. jejuni* antimicrobial drug resistance have been considered extremely low; during 2000–2013, prevalence of fluoroquinolone resistance in *Campylobacter* spp. isolated from humans was reportedly <6% (*36*). During 2005–2006, no resistance to tetracyclines or fluoroquinolones was found in 193 *C. jejuni* isolates from poultry (*37*); a 2009 systematic survey of antimicrobial drug resistance in animal (calves and poultry) isolates of *C. jejuni* found no resistance to erythromycin, 0.3% resistance to tetracycline, and only 2.3% resistance to fluoroquinolones (*38*). The emergence of this lineage is unlikely to be the result of fluoroquinolone use in the food chain because the poultry industry in New Zealand does not use fluoroquinolones (*39*,*40*).

Our data demonstrate the utility of systematic phenotypic surveillance of antimicrobial drug resistance in *C. jejuni,* which is becoming increasingly necessary as laboratories adopt routine culture-independent diagnostic testing for enteric pathogens. The use of phenotypic surveillance is particularly relevant for *Campylobacter*, for which culture-independent diagnostic testing is replacing culture-based diagnosis in many settings (*41**,**42*). Although recent whole-genome sequencing–based work demonstrated good concordance between antimicrobial-resistance genotype and phenotype in *Campylobacter* spp. (*43*), uncharacterized mutations are unlikely to be detected and isolates are still required for whole-genome sequencing analysis. To ensure ongoing culture capability and the capacity to undertake periodic phenotypic antimicrobial-resistance testing, close liaison between clinical and public health laboratories is needed.

Both the *tetO* gene and the prophage-integrated element CJIE1v may have originated on the remnant plasmid and been inserted into the genome of ST6964. One possible scenario is that the common ancestor of ST6964 acquired a plasmid similar to megaplasmid pcjDM, which carried the *tetO* gene and a phage. Under this scenario, the *tetO* gene was then inserted into the genome at a single site and the phage element was inserted into multiple sites, leaving the remnant plasmid with the backbone minus the *tetO* and CJIE1v sequences. We propose that the remnant plasmid was then lost from a common ancestor of isolates in poultry company C (Figure 3).

Although the *tetO* flanking genes in the chromosome differ from the *tetO* cargo in megaplasmid pcjDM, evidence that these came from the plasmid comes from isolate 15AR1747, which contains additional chromosomal genes adjacent to the *tetO* sequence that are identical to those found in the remnant plasmid of all other plasmid-bearing *C. jejuni* ST6964 isolates. Furthermore, these genes are absent from the smaller remnant plasmid identified in 15AR1747 (Appendix Figure 6). 

Both CJIE1 and CJIE4 are prophages (*29*). CJIE1 has been associated with increased adherence and invasion (*44*) and differences in protein expression under different conditions (*45*). The multiple locations of prophage-integrated element CJIE1 has been identified in previous studies (*29*). Previous studies have shown that both CJIE1 and CJIE4 encode nucleases that hydrolyze DNA and inhibit natural transformation (*46**,**47*). Prophage-integrated elements in addition to the plasmid may have played some role in the evolution of ST6964 in New Zealand, potentially stabilizing lineages by reducing transformability (*47*); however, what may have influenced their frequency and distribution among poultry companies and hosts is unclear.

## Conclusions

The emergence of antimicrobial-drug resistant *C. jejuni* ST6964 in New Zealand poultry and transmission to humans via the food chain underlines the role of the fresh poultry supply as a source of human cases of campylobacteriosis and how rapidly new clones can evolve and spread. We provide evidence that this clone has undergone rapid evolution in New Zealand through multiple mechanisms, including mutations/substitutions, conjugation, natural transformation, and the incorporation of prophages into the chromosome. Given its speed of emergence and its spread across vertically integrated poultry companies, it is imperative that ongoing periodic surveillance of antimicrobial drug resistance in *Campylobacter* and other relevant bacterial pathogens is supported by government agencies to better track the emergence and possible further spread of resistance in New Zealand. This surveillance includes gathering information at the farm level to determine the relative roles of different transmission pathways that could account for spread within and between poultry companies.

Ongoing work indicates that *C. jejuni* ST6964 is persisting in the poultry supply and continuing to make a considerable contribution to the country’s disease burden. This finding has implications for the use of antimicrobial drugs; for example, fluoroquinolones are likely to be ineffective for treatment of severe and invasive campylobacteriosis. To control and mitigate the spread of this clone, appropriate source control measures and increased public awareness of appropriate food hygiene should be considered by the government and the poultry industry, along with the development of rapid, less costly diagnostic assays, which could be facilitated by data derived from whole-genome sequencing.

Appendix Supplemental information for genomic analysis of fluoroquinolone- and tetracycline-resistant *Campylobacter jejuni* sequence type 6964 in humans and poultry, New Zealand, 2014–2016.
