# Supplementary material for: Genomic Analysis of Fluoroquinolone- and Tetracycline-Resistant Campylobacter jejuni Sequence Type 6964 in Humans and Poultry, New Zealand, 2014–2016
Source: Emerg Infect Dis. 2019 Dec;25(12):2226–34. doi: 10.3201/eid2512.190267 (PMC6874264; doi:10.3201/eid2512.190267)
Supplement: Appendix — Supplemental information for genomic analysis of fluoroquinolone- and tetracycline-resistant Campylobacter jejuni sequence type 6964 in humans and poultry, New Zealand, 2014–2016. [file 19-0267-Techapp-s1.pdf]

Article DOI: https://doi.org/10.3201/eid2512.190267

Genomic Analysis of Fluoroquinolone- and Tetracycline-Resistant *Campylobacter jejuni* Sequence Type 6964 in Humans and Poultry, New Zealand, 2014–2016

Appendix

Further details of participating human diagnostic laboratories and poultry farm survey

The laboratories that referred isolates for the human case survey were Labtests, Auckland; Pathlab Bay of Plenty; Aotea Pathology, Wellington; Canterbury Southern Community Laboratories, Christchurch; and Southern Community Laboratories, Dunedin. Details of the survey are provided are provided in the report by Williamson, Dyet (*1*).

For the poultry cecal surveys pooled cecal samples (each containing cecal contents from 5 chickens) were taken from 4 poultry companies at slaughter. One study was conducted between May 25, 2015 and June 02, 2015 and the other between July 07, 2015 and March 25, 2016. A total of 897 pooled cecal samples from broiler and breeder flocks were processed, of which 53.7% (482/897) were positive for growth typical of *Campylobacter* on selective media containing ciprofloxacin and tetracycline. Only six composite samples were received from the small Company D and none of these samples yielded growth on the mCCDA + cip/tet plates. A random subset of 291 isolates were speciated by PCR and all were confirmed as *C. jejuni*. A further subset of 99 of the 291 isolates were subtyped by 7-gene MLST and all were confirmed as ST-6964. Given the high probability that isolates cultured on the selective media were *C. jejuni* ST-6964, 136 of the 291 PCR confirmed isolates (from 118 pooled samples) were randomly selected for WGS, and all were confirmed by WGS as ST-6964 (39 by 7-gene MLST and WGS and 97 by WGS only).

A map showing the location of the participating laboratories and the poultry farms from which *C. jejuni* ST 6964 positive cecal samples were taken for analysis is shown in Appendix Figure 1.

References

1. Williamson D, Dyet K, Heffernan H. Antimicrobial resistance in human isolates of *Campylobacter jejuni*, 2015: Institute of Environmental Science and Research Limited; 2015.

2. Marasini D, Fakhr MK. Whole-genome sequencing of a *Campylobacter jejuni* strain isolated from retail chicken meat reveals the presence of a megaplasmid with Mu-like prophage and multidrug resistance genes. Genome Announc. 2016;4:e00460-16. PubMed https://doi.org/10.1128/genomeA.00460-16

3. Marasini D, Fakhr MK. Complete genome sequences of plasmid-bearing multidrug-resistant *Campylobacter jejuni* and *Campylobacter coli* strains with type VI secretion systems, isolated from retail turkey and pork. Genome Announc. 2017;5:e01360-17. PubMed https://doi.org/10.1128/genomeA.01360-17

4. Marasini D, Fakhr MK. Complete genome sequences of *Campylobacter jejuni* strains isolated from retail chicken and chicken gizzards. Genome Announc. 2017;5:e01351-17. PubMed https://doi.org/10.1128/genomeA.01351-17

5. Crespo MD, Altermann E, Olson J, Miller WG, Chandrashekhar K, Kathariou S. Novel plasmid conferring kanamycin and tetracycline resistance in the turkey-derived *Campylobacter jejuni* strain 11601MD. Plasmid. 2016;86:32–7. PubMed https://doi.org/10.1016/j.plasmid.2016.06.001

6. Gunther NW IV, Reichenberger ER. Complete genome sequence of *Campylobacter jejuni* RM1246-ERRC, which exhibits resistance to quaternary ammonium compounds. Genome Announc. 2017;5:e00978-17. PubMed https://doi.org/10.1128/genomeA.00978-17

7. Batchelor RA, Pearson BM, Friis LM, Guerry P, Wells JM. Nucleotide sequences and comparison of two large conjugative plasmids from different *Campylobacter* species. Microbiology. 2004;150:3507–17. PubMed https://doi.org/10.1099/mic.0.27112-0

8. Marasini D, Fakhr MK. Complete genome sequences of *Campylobacter jejuni* strains OD267 and WP2202 isolated from retail chicken livers and gizzards reveal the presence of novel 116-Kilobase and 119-Kilobase megaplasmids with type VI secretion systems. Genome Announc. 2016;4:e01060-16. PubMed https://doi.org/10.1128/genomeA.01060-16

9. Taveirne ME, Dunham DT, Perault A, Beauchamp JM, Huynh S, Parker CT, et al. Complete annotated genome sequences of three *Campylobacter jejuni* strains isolated from naturally colonized farm-raised chickens. Genome Announc. 2017;5:e01407-16. PubMed https://doi.org/10.1128/genomeA.01407-16

10. Zhang M, He L, Li Q, Sun H, Gu Y, You Y, et al. Genomic characterization of the Guillain-Barre syndrome-associated *Campylobacter jejuni* ICDCCJ07001 Isolate. PLoS One. 2010;5:e15060. PubMed https://doi.org/10.1371/journal.pone.0015060

11. Cooper KK, Cooper MA, Zuccolo A, Law B, Joens LA. Complete genome sequence of *Campylobacter jejuni* strain S3. J Bacteriol. 2011;193:1491–2. PubMed https://doi.org/10.1128/JB.01475-10

Appendix Table 1. Details of the plasmids used in the comparative analysis of plasmid 15AR0984-m from the reference strain of ST-6964 used in this study

Accession no.	Reference	Description
CP013117.1	(*2*)	*C. jejuni* strain T1–21 plasmid pcjDM
CP017857.1	(*3*)	*C. jejuni* strain YQ2210 plasmid pCJDM210L
CP017854.1	(*4*)	*C. jejuni* strain ZP3204 plasmid pCJDM204L
KJ646012.1	(*5*)	*C. jejuni* strain 11601MD plasmid p11601MD
CP022471.1	(*6*)	*C. jejuni* strain RM1246-ERRC plasmid pRM1246_ERRC
AY394561.1	(*7*)	*C. jejuni* strain 81–176 plasmid pTet
CP014745.1	(*8*)	*C. jejuni* strain OD267 plasmid pCJDM67 L
CP017418.1	(*9*)	*C. jejuni* strain MTVDSCj13 plasmid pMTVDSCj13–1
CP007750.1	Not applicable	*C. jejuni* strain M129 plasmid pTet-M129
CP007182.1	Not applicable	*C. coli* strain RM4661 plasmid pRM4661
CP002030.1	(*10*)	*C. jejuni* strain ICDCCJ07001 plasmid pTet
CP001961.1	(*11*)	*C. jejuni* strain S3 plasmid pTet

Appendix Table 2. The 87 wgMLST shared-loci that were within the Gubbins predicted recombination regions. The aliases in the first column correspond to the gene names used in the wgMLST analysis.

Alias	Gene	Function	Locus tag
Gene577		Hypothetical protein	15AR0984_00592
Gene1430		Peptidylprolyl isomerase	15AR0984_01465
Gene1431	*accC*	Biotin carboxylase	15AR0984_01466
Gene1432	*accB*	Biotin carboxyl carrier protein of acetyl-coa carboxylase	15AR0984_01467
Gene1433	*dcd*	Deoxycytidine triphosphate deaminase	15AR0984_01468
Gene1434	*pseB*	UDP-N-acetylglucosamine 4,6-dehydratase (inverting)	15AR0984_01469
Gene1435	*pseC*	UDP-4-amino-4, 6-dideoxy-N-acetyl-β-L-altrosamine transaminase	15AR0984_01470
Gene1437		Putative polysaccharide biosynthesis protein with aminopeptidase-like domain protein	15AR0984_01472
Gene1441		Hypothetical protein	15AR0984_01476
Gene1442	*fabH*	3-oxoacyl-[acyl-carrier-protein] synthase 3	15AR0984_01477
Gene1443		Hypothetical protein	15AR0984_01478
Gene1444		Hypothetical protein	15AR0984_01479
Gene1445		Hypothetical protein	15AR0984_01480
Gene1449		Hypothetical protein	15AR0984_01484
Gene1454	*legF*	CMP-N,N'-diacetyllegionaminic acid synthase	15AR0984_01489
Gene1455	*pseG*	UDP-2,4-diacetamido-2,4, 6-trideoxy-β-L-altropyranose hydrolase	15AR0984_01490
Gene1456	*pseH*	UDP-4-amino-4, 6-dideoxy-N-acetyl-β-L-altrosamine N-acetyltransferase	15AR0984_01491
Gene1457	*hisF*	Imidazole glycerol phosphate synthase subunit hisf	15AR0984_01492
Gene1458	*hisH1*	Imidazole glycerol phosphate synthase subunit hish 1	15AR0984_01493
Gene1459		Hypothetical protein	15AR0984_01494
Gene1461	*rmlB*	Dtdp-glucose 4,6-dehydratase	15AR0984_01496
Gene1464	*legI*	N,N'-diacetyllegionaminic acid synthase	15AR0984_01499
Gene1469	*hddC*	D-glycero-α-D-manno-heptose 1-phosphate guanylyltransferase	15AR0984_01504
Gene1470		Hypothetical protein	15AR0984_01505
Gene1471	*legF*	CMP-N,N'-diacetyllegionaminic acid synthase	15AR0984_01506
Gene1472	*lvr*	Levodione reductase	15AR0984_01507
Gene1476		Hypothetical protein	15AR0984_01511
Gene1481		Hypothetical protein	15AR0984_01516
Gene1482		Hypothetical protein	15AR0984_01517
Gene1483	*xcpT*	Type II secretion system protein G	15AR0984_01518
Gene1484	*tsaD*	Trna N6-adenosine threonylcarbamoyltransferase	15AR0984_01519
Gene1486	*dxr*	1-deoxy-D-xylulose 5-phosphate reductoisomerase	15AR0984_01521
Gene1487	*cdsA*	Phosphatidate cytidylyltransferase	15AR0984_01522
Gene1488		Hypothetical protein	15AR0984_01523
Gene1489		Hypothetical protein	15AR0984_01524
Gene1491		Putative phospholipase A1	15AR0984_01526
Gene1492	*feuB*	Iron-uptake system permease protein feub	15AR0984_01527
Gene1493	*feuC*	Iron-uptake system permease protein feuc	15AR0984_01528
Gene1494	*fhuC*	Iron(3+)-hydroxamate import ATP binding protein fhuc	15AR0984_01529
Gene1496		Hypothetical protein	15AR0984_01532
Gene1497	*nrfA*	Cytochrome c-552	15AR0984_01533
Gene1498	*nrfH*	Cytochrome c-type protein nrfh	15AR0984_01534
Gene1499	*ppk*	Polyphosphate kinase	15AR0984_01535
Gene1504		Hypothetical protein	15AR0984_01541
Gene1505	*ruvB*	Holliday junction ATP-dependent DNA helicase ruvb	15AR0984_01542
Gene1507	*fumC*	Fumarate hydratase class II	15AR0984_01544
Gene1509	*glmS*	Glutamine–fructose-6-phosphate aminotransferase [isomerizing]	15AR0984_01546
Gene1511	*mqnE*	Aminodeoxyfutalosine synthase	15AR0984_01548
Gene1512	*adeQ*	Adenine permease adeq	15AR0984_01549
Gene1513	*pyrE*	Orotate phosphoribosyltransferase	15AR0984_01550
Gene1514	*mlaA*	Putative phospholipid binding lipoprotein mlaa	15AR0984_01551
Gene1515	*mlaC*	Putative phospholipid binding protein mlac	15AR0984_01552
Gene1516		Bifunctional preprotein translocase subunit secd/secf	15AR0984_01553
Gene1589	*kpsM*	Polysialic acid transport protein kpsm	15AR0984_01626
Gene1590		Hypothetical protein	15AR0984_01627
Gene1591		Hypothetical protein	15AR0984_01628
Gene1592		Dutpase	15AR0984_01629
Gene1593		Hypothetical protein	15AR0984_01630
Gene1595	*rimO*	Ribosomal protein S12 methylthiotransferase rimo	15AR0984_01632
Gene1596	*prfB*	Peptide chain release factor 2	15AR0984_01633
Gene1597		Hypothetical protein	15AR0984_01634
Gene1598	*truD*	Trna pseudouridine synthase D	15AR0984_01635
Gene1599	*thiL*	Thiamine-monophosphate kinase	15AR0984_01636
Gene1601		Hypothetical protein	15AR0984_01638

Alias	Gene	Function	Locus tag
Gene1602	*rsmD*	Ribosomal RNA small subunit methyltransferase D	15AR0984_01639
Gene1603	*flgI*	Flagellar P-ring protein	15AR0984_01640
Gene1604		Hypothetical protein	15AR0984_01641
Gene1605		Anti-sigma-28 factor, flgm	15AR0984_01642
Gene1606		Flgn protein	15AR0984_01643
Gene1607	*flgK*	Flagellar hook-associated protein 1	15AR0984_01644
Gene1609		Hypothetical protein	15AR0984_01646
Gene1616	*por*	Pyruvate synthase	15AR0984_01653
Gene1617	*ppaX*	Pyrophosphatase ppax	15AR0984_01654
Gene1618	*oprF*	Outer membrane porin F	15AR0984_01655
Gene1619	*rpsI*	30S ribosomal protein S9	15AR0984_01656
Gene1620	*rplM*	50S ribosomal protein L13	15AR0984_01657
Gene1621	*addA*	ATP-dependent helicase/nuclease subunit A	15AR0984_01658
Gene1624		Hypothetical protein	15AR0984_01661
Gene1625		Hypothetical protein	15AR0984_01662
Gene1626		Hypothetical protein	15AR0984_01663
Gene1627	*fixP*	Cbb3-type cytochrome c oxidase subunit fixp	15AR0984_01664
Gene1628		Cbb3-type cytochrome oxidase component fixq	15AR0984_01665
Gene1629		Cytochrome C oxidase, mono-heme subunit/fixo	15AR0984_01666
Gene1630	*ccoN1*	Cbb3-type cytochrome c oxidase subunit ccon1	15AR0984_01667
Gene1631	*mprA*	Response regulator mpra	15AR0984_01668
Gene1640		Hypothetical protein	15AR0984_01678
Gene1641	*putP*	Sodium/proline symporter	15AR0984_01679

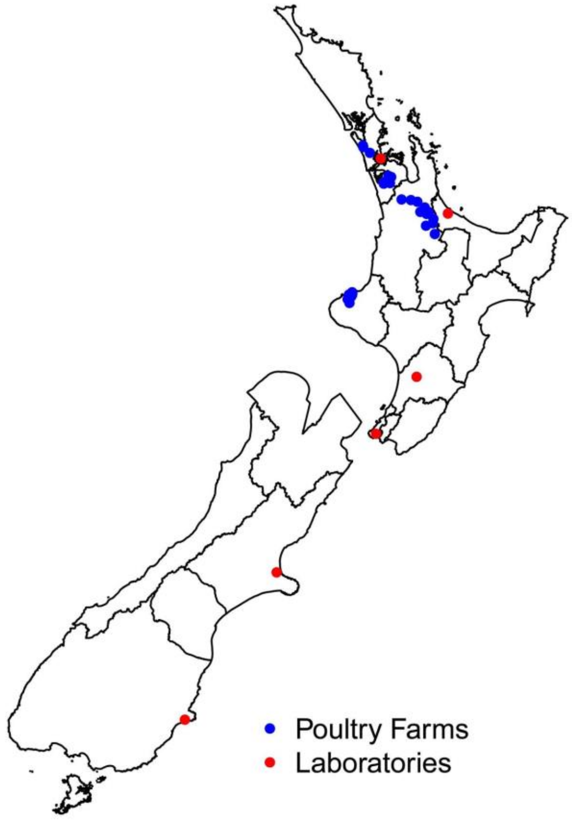

Appendix Figure 1. The location of the poultry farms from which *C. jejuni* ST 6964 positive cecal samples were taken and the diagnostic laboratories that submitted human isolates including the 5 that participated in the human survey and the MidCentral laboratory in the Manawatu sentinel site.

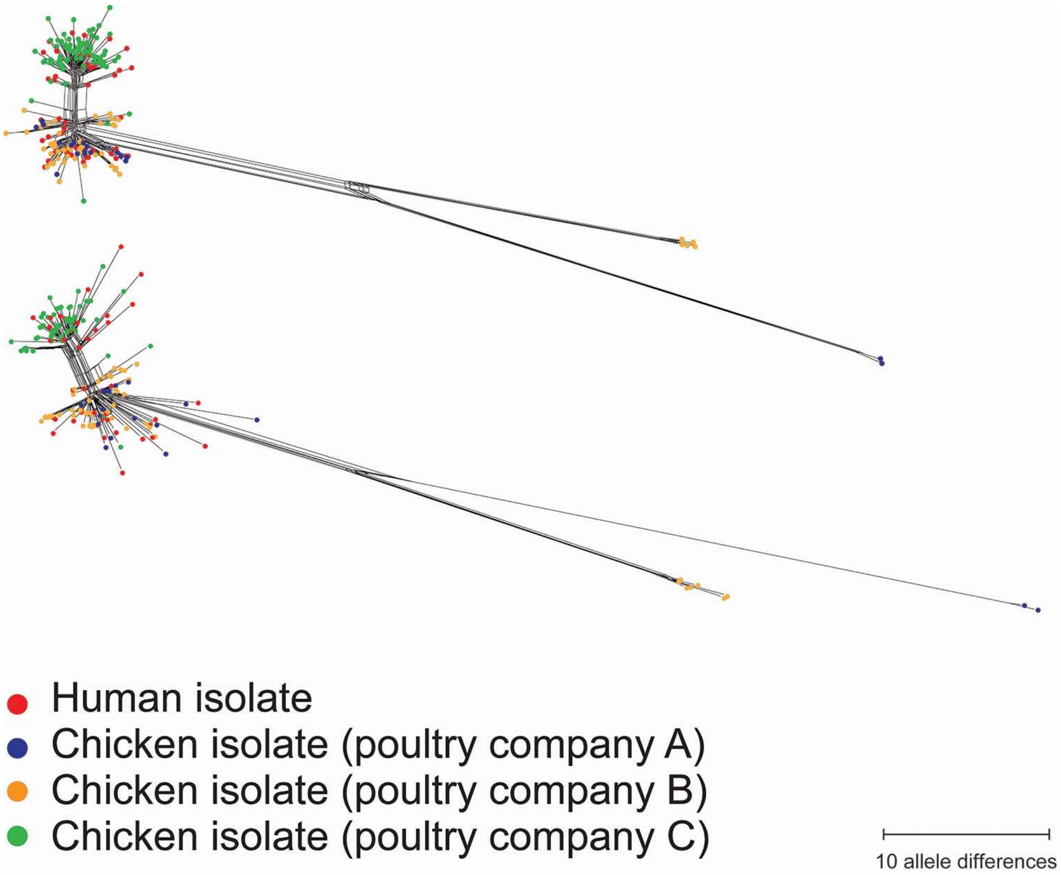

Appendix Figure 2. Pairwise distances and NeighborNet networks of the 227 *C. jejuni* ST-6964 isolates calculated by two independent wgMLST methods. The upper network was generated from the 1,363 shared loci found in the ad hoc wgMLST analysis; and the lower network was generated with the *C. jejuni* cgMLST scheme (1,343 loci) on the PubMLST Web site.

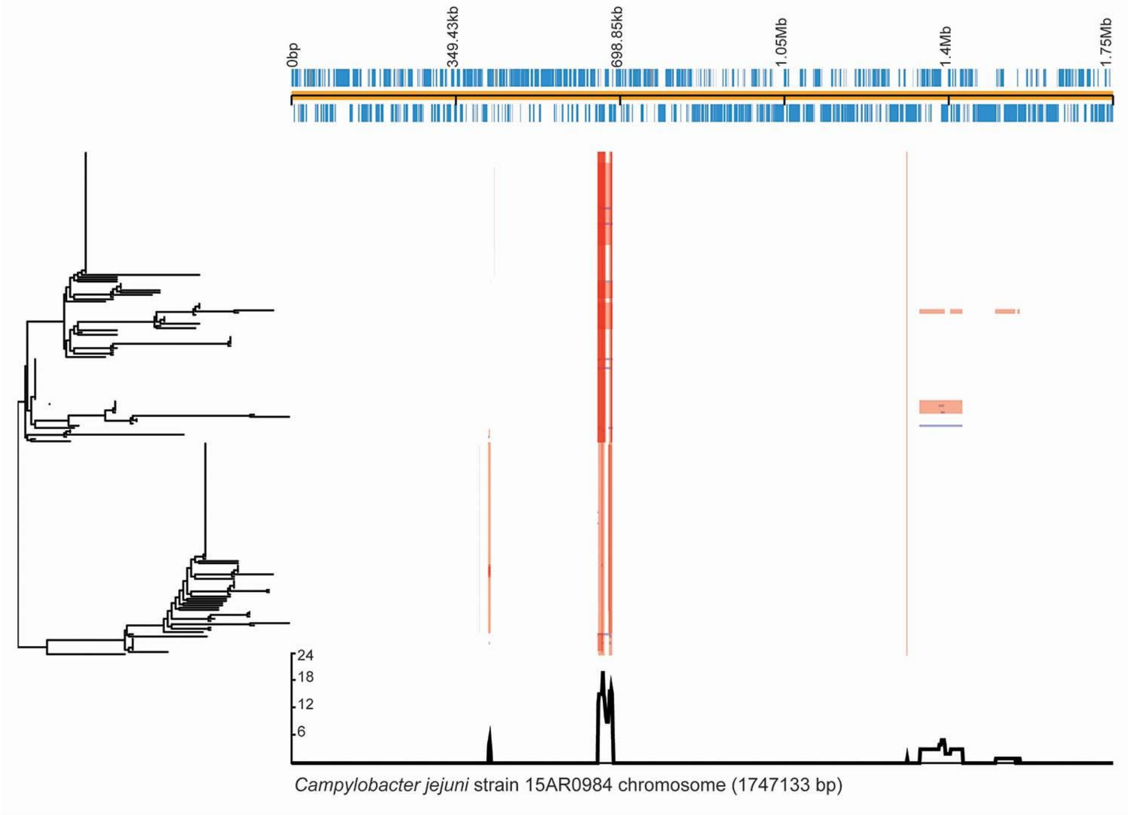

Appendix Figure 3. Recombination detected in 227 *C. jejuni* ST-6964 isolates. Coding regions are shown on both the forward and reverse strand of the annotated *C. jejuni* 15AR0984 reference genome. The tree is the inferred mid-point rooted phylogeny of 227 *C. jejuni* ST-6964 isolates and reference 15AR0984 genome is shown to the left and the recombination blocks identified by Gubbins are shown to the right of the tree; red indicates conserved blocks, blue indicates blocks detected in just one representative. The lower plot (black line) summarizes the number of recombination events across the entire *C. jejuni* reference genome.

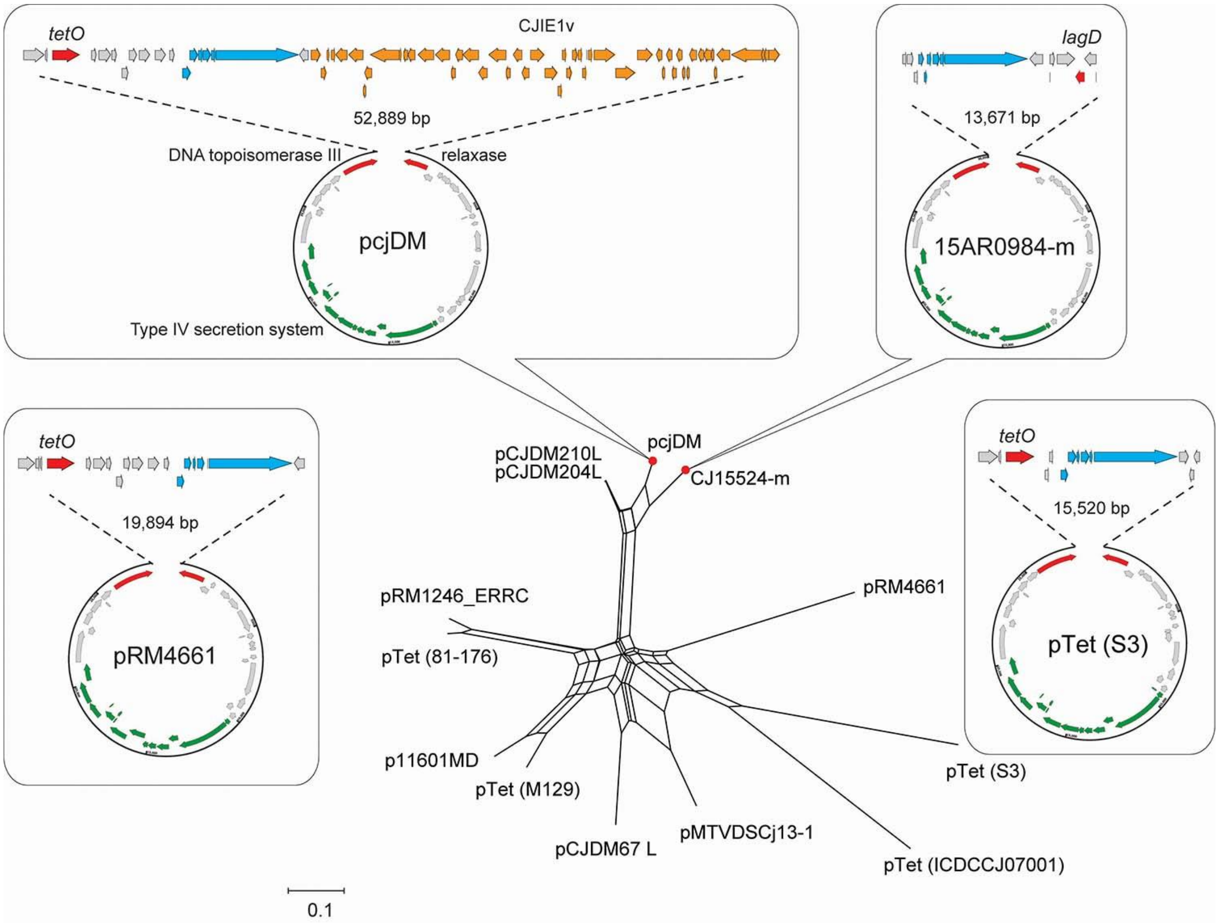

Appendix Figure 4. NeighborNet phylogenetic relationship of the plasmid identified in the complete genome of isolate 15AR0984 related to nine other representative 'pTet-like' plasmids, based on the allele profiles of the 23 shared-loci. The genome structures of the closest plasmid pcjDM (four allele differences) and the other three plasmids were plotted to demonstrate the consensus regions (backbone) and hyper-variable regions in the plasmids.

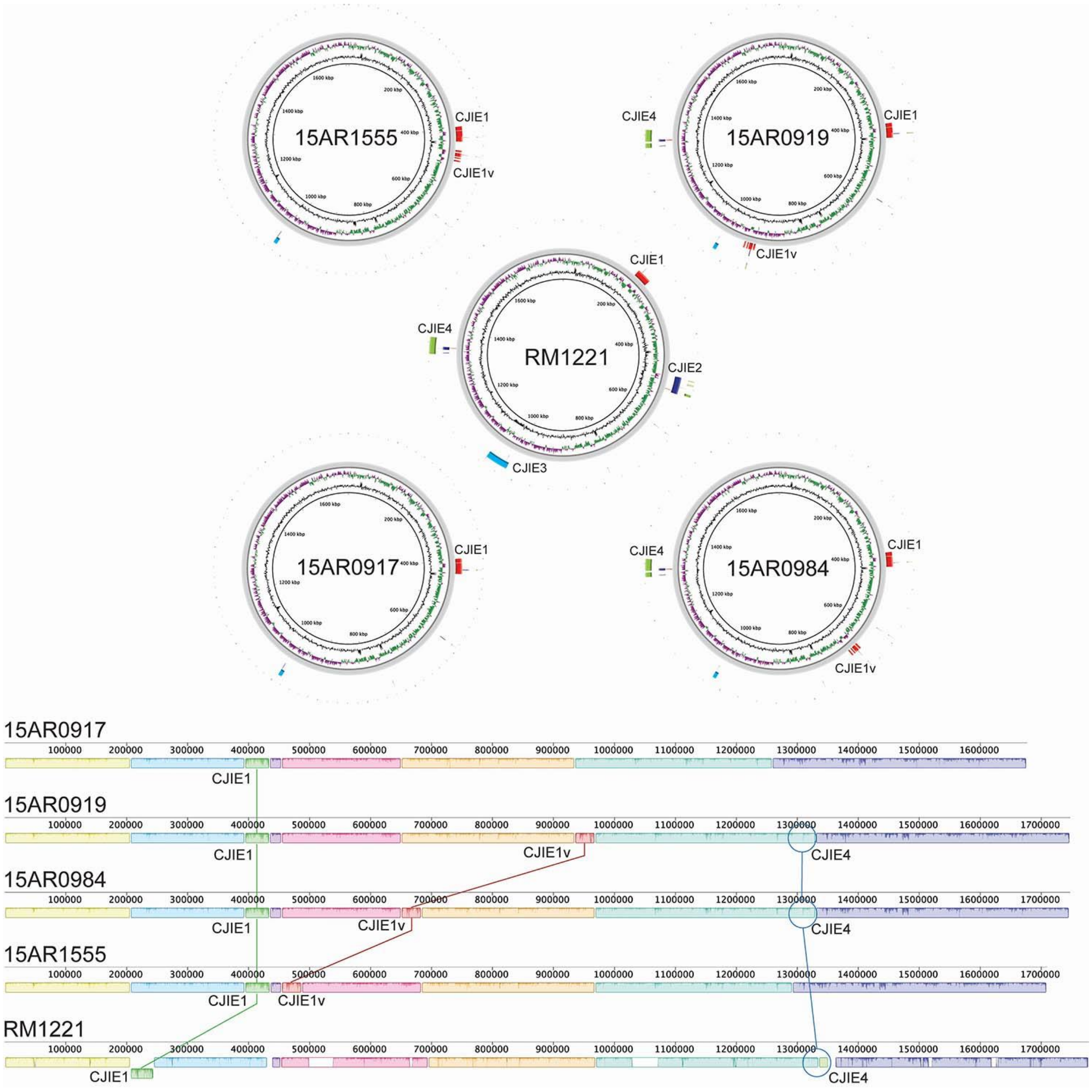

Appendix Figure 5. The insertion locations of mobile elements CJIE1, CJIE1v and CJIE4 are illustrated in a whole genome alignment of the four complete genomes of ST-6964 isolates (15AR0984, 15AR0917, 15AR0919 and 15AR1555) and the reference RM1221. The upper rings were generated with the BLAST Ring Image Generator (BRIG) and the lower linear plots with Mauve. The mobile element CJIE1 was present in all isolates. CJIE1v was inserted in the three different locations in three of the ST-6964 isolate genomes.

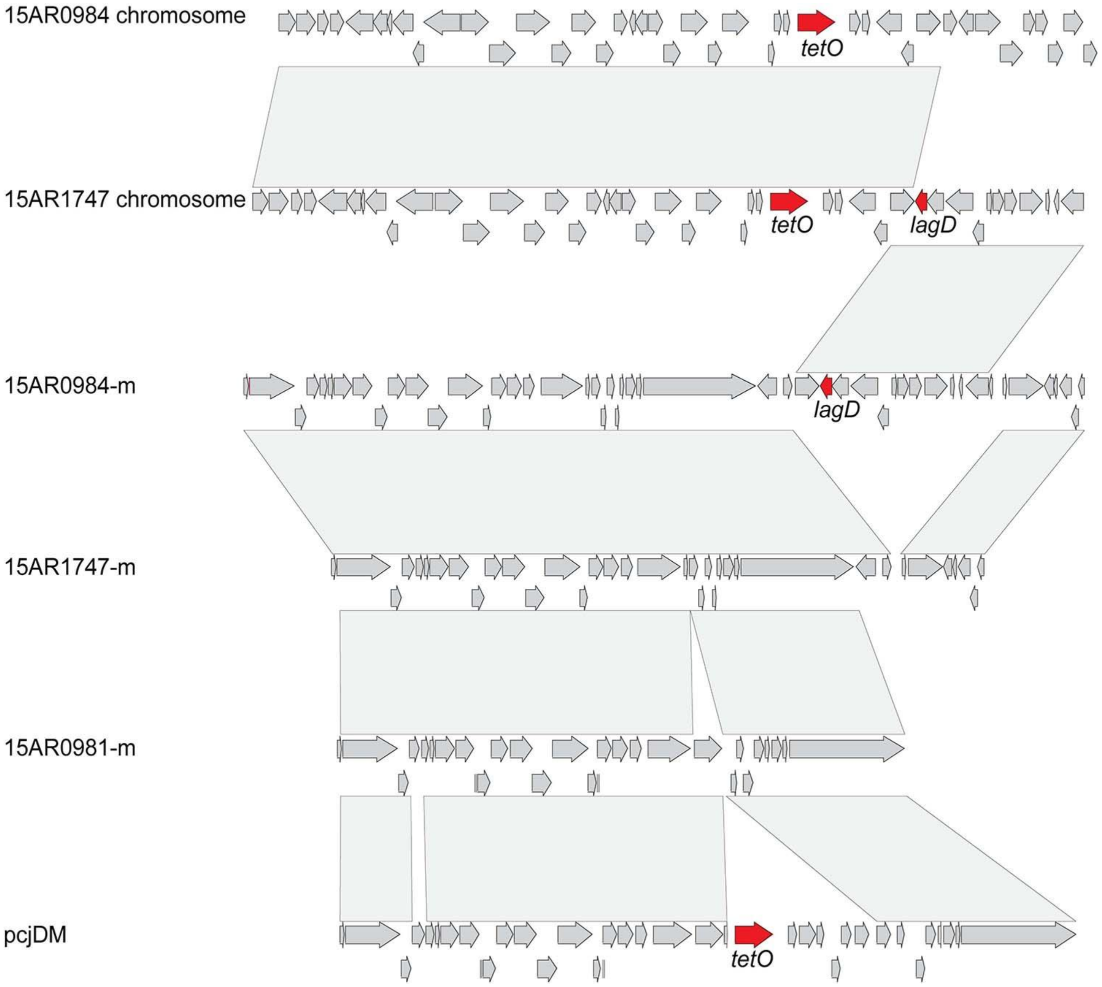

Appendix Figure 6. High-scoring Segment Pairs (HSPs) between the chromosome and plasmid sequences of the strain 15AR0984, 15AR1747, 15AR0981 and T1–21 (pcjDM) were connected with gray bars to demonstrate similar regions. We hypothesize the *lagD* and *tetO* genes were in the same region and located in the plasmid of the ST-6964 ancestor strain, and the genome structure of the region in the ancestral plasmid was similar to the equivalent region in the chromosome of isolate 15AR1747. Subsequently, we hypothesize, this region was excised from the 15AR1747-m plasmid and integrated into its chromosome; whereas in 15AR0984-m plasmid, only the *tetO* gene region was excised and integrated into its chromosome, leaving *lagD* still in the 15AR0984-m plasmid. Alternatively, the *tetO* could be carried by a pcJDM-like plasmid, and subsequently excised from the 15AR0981-m plasmid and integrated the chromosomes (15AR0984 and 15AR1747 chromosomes).
